# Peak Detection with Online Electroencephalography (EEG) Artifact Removal for Brain–Computer Interface (BCI) Purposes

**DOI:** 10.3390/brainsci9120347

**Published:** 2019-11-29

**Authors:** Mihaly Benda, Ivan Volosyak

**Affiliations:** Faculty of Technology and Bionics, Rhine-Waal University of Applied Sciences, 47533 Kleve, Germany

**Keywords:** Brain-Computer Interface (BCI), Steady-State Visual Evoked Potential (SSVEP), artefact removal, Individual Alpha Peak, movement artefact, Electroencephalography (EEG)

## Abstract

Brain–computer interfaces (BCIs) measure brain activity and translate it to control computer programs or external devices. However, the activity generated by the BCI makes measurements for objective fatigue evaluation very difficult, and the situation is further complicated due to different movement artefacts. The BCI performance could be increased if an online method existed to measure the fatigue objectively and accurately. While BCI-users are moving, a novel automatic online artefact removal technique is used to filter out these movement artefacts. The effects of this filter on BCI performance and mainly on peak frequency detection during BCI use were investigated in this paper. A successful peak alpha frequency measurement can lead to more accurately determining objective user fatigue. Fifteen subjects performed various imaginary and actual movements in separate tasks, while fourteen electroencephalography (EEG) electrodes were used. Afterwards, a steady-state visual evoked potential (SSVEP)-based BCI speller was used, and the users were instructed to perform various movements. An offline curve fitting method was used for alpha peak detection to assess the effect of the artefact filtering. Peak detection was improved by the filter, by finding 10.91% and 9.68% more alpha peaks during simple EEG recordings and BCI use, respectively. As expected, BCI performance deteriorated from movements, and also from artefact removal. Average information transfer rates (ITRs) were 20.27 bit/min, 16.96 bit/min, and 14.14 bit/min for the (1) movement-free, (2) the moving and unfiltered, and (3) the moving and filtered scenarios, respectively.

## 1. Introduction

Electroencephalography (EEG) recordings of brain activity are used for multiple purposes, from medical diagnostics to brain–computer interfaces (BCIs) [1,2]. The recorded activity is analysed with various methods according to the end goal, and one or several features are extracted and interpreted.

BCIs evaluate specific components of brain activity and try to classify them according to criteria set previously, in order to execute a corresponding command when such a component is detected. By providing multiple types of commands, the control of a BCI system can be handled by the users’ brain activity alone (interpreted by the BCI) without the need for muscle movements. Steady-state visual evoked potentials (SSVEPs) are one of the specific brain activities which BCIs can utilise. They are generated when the user is looking at a source of light that flickers with a constant frequency (for example, by changing colour or luminance). BCI performance can be easily influenced by the recording environment. The lighting conditions in the room, movements of the users, or even the attention and fatigue levels significantly alter the speed of the BCI.

User fatigue could be partially or even totally countered by changing the parameters of the BCI. For this, a measure of the subjects’ fatigue is necessary. Although there are subjective methods for acquiring fatigue levels from the users, for example, questionnaires, there are two main drawbacks to these methods. The subjectivity can cause mistakes in the following parameter changes, possibly not improving the BCI performance at all, or even detrimentally affecting it. Secondly, these methods require the users to stop using the BCI and, for example, fill out a questionnaire. As BCIs are online systems taking breaks during their use does not make sense. Therefore an online and objective measure is required for optimal use with BCIs.

EEG recordings are also commonly utilised in the research of brain activity and functions [3,4]. For example alpha activity can be associated with attention [5]. Alpha peaks and mean powers of different frequency bands have been proposed to indicate user attention or tiredness during BCI use [6,7,8,9]. The reported results of these investigations vary and are sometimes contradicting.

Moreover, there are findings in the field of neurosciences, which were not considered by the previously mentioned researches for measuring fatigue associated with BCIs. Namely, the inter-individual differences, how these affect the bandwidths, already discussed in [10], and the methods for determining the peak frequency in a band [11]. The traditional band limits may be inappropriate, as there are differences between the subjects to such an extent that their peak alpha frequency, for example, may fall entirely out of the traditional alpha range. Although this will not affect finding the peak in most cases, as it is still likely within the range, the mean power or any ratio calculated from it will be affected, however. To determine the individual peaks, a robust and accurate method is required, which can handle the common pitfalls of peak detection, such as split peaks.

There are several different techniques, with benefits and drawbacks, that have been applied for determining peak frequencies. They include simple and complicated methods alike [11]. One of the most common methods is specifying the frequency band, by extracting data from the fast Fourier transform (FFT), which is limited by traditional, personalised, or in some way generalised frequency borders (e.g., 8–13 Hz for alpha band), and finding the local maximum. Determining the peak alpha frequency in a condition where the eyes are closed is usually straightforward; however, with open eyes this task can become quite challenging. If there are no obvious peaks in the range, or there is a split peak, the estimation can become biased and incorrect [11]. Since visual BCIs require open eyes, this method is not suitable for peak detection during SSVEP-based BCI use.

An alternative method of determining the highest peak in a frequency band was developed originally by [12]. The estimation is done by calculating a weighted average of the power contained in the frequency band that is sensitive to the spectral distribution. This method can yield results even if a clear peak is not detectable. However, it requires a clear definition of the boundaries of the band. Since brain activity has a high inter-subject variance, the boundaries cannot be defined in a generalised way without somehow biasing the estimation. The behaviour of the frequency components of the recorded brain activity can also be profoundly different. This makes the estimation of the peak frequency challenging to automate, even when utilising data recorded with different conditions (e.g., eyes open and closed) [11].

A less commonly used method is curve fitting, which can alleviate several of the previous issues [13,14]. The problems split peaks have caused can be alleviated, for example, and the boundaries play a lesser role in finding the peak frequency. A curve fitting method for finding peak alpha frequencies was introduced by [13] and was improved later to account for split peaks [15].

A promising and already implemented approach with curve fitting is provided by [11], who utilises a Savitzky–Golay filter for the smoothing of the FFT, which can ease peak detection. It also includes calculating the derivatives of the FFT spectrum and finding inflexion points before applying curve fitting based on the least-squares approach. Savitzky–Golay filters have the important property of not altering the properties of the peaks. In this experiment this method is used for individual alpha frequency (IAF) detection during simple EEG recordings and SSVEP-based BCI tasks.

With the individual alpha frequency (IAF), the individual alpha ranges can be defined, and phase information can be calculated, which seem to be promising approaches to measuring attention or fatigue. They can show whether the target brain area is receiving external information from the stimuli, or if the information gathering is blocked by the alpha activity [5,16].

There is another issue to consider when using BCIs and neural activity measurements, which is the artefacts present in the recorded EEG data. All previously mentioned peak detection methods can suffer from ocular or muscular artefacts, which can easily occur during a recording. With all different methods of peak estimation, it is crucial to have data with a high signal to noise ratio (SNR), or preferably, without any noise. Most of the studies handling EEG data, to minimise noise, require the subjects to sit in a comfortable position and move as little as possible, or even physically fix the position of the head by using head-mounts.

However, BCIs aim to develop solutions for people with disabilities or for use in everyday scenarios, and as such they have to consider head movements and blinking, which are unavoidable for any prolonged use of the system. This is especially true with mobile systems, as more movements are expected if the wires do not restrict the users. Commonly offline analysis is used after rejecting all noisy data from further calculations, however, this cannot be done with BCI systems. BCIs require online and therefore (semi-)automatic artefact removal and analysis. Optimally BCI systems require an artefact correction (and not rejection) method, in order to always be able to process the users’ EEG activity. The exact choice of artefact removal algorithm is not simple. These usually remove one or both of the most common EEG artefacts, eye movements, and muscle movements.

The authors in [17] presented a method based on correlation index and the feature of power distribution to automatically detect eye blink components, using extended infomax ICA. Ref. [18] presented a way of removing ocular artefacts by using blind source separation on the raw EEG recording.

Regarding muscular artefacts, Ref. [19] demonstrated muscular artefact cancellation in single-channel EEG recordings with a combination of ensemble empirical mode decomposition and joint blind source separation. They successfully removed muscular artefacts without altering the underlying EEG activity.

More recently, Ref. [20] introduced the source-estimate-utilizing noise-discarding (SOUND) algorithm. It uses Wiener estimators and employs anatomical information to identify and suppress noise and artefacts in EEG and MEG. Also recently, Ref. [21] developed a generic EEG artefact removal algorithm, which allows the annotation of artefact segments and clear segments. With this information the algorithm based on the multi-channel Wiener filter (MWF) can remove a wide variety of artefacts from EEG data. The result was a high-performance semi-automatic artefact removal algorithm able to remove both muscular and ocular artefacts. This algorithm requires only a short time to do computations and handles both common EEG artefact types—ocular and muscular—therefore, it is optimal for use with BCIs. Authors in [21] tested their algorithm performance using both hybrid and real EEG recordings. However, the calculations were offline, and the selection of the artefact segments is made manually by the experimenter. For this experiments’ purpose the calculations had to be done online. Moreover, the effect of such a filter on the measurement of peak frequencies from an FFT transformed signal needed to be investigated.

In this experiment, we wished to create a scenario that could easily occur when using a mobile BCI system. Therefore, the experiment necessitated movements which would occur in everyday scenarios, such as chewing, speaking, and head gestures, freely selected and even combined by the users. We then used the automatised version of the presented artefact removal algorithm in online EEG recordings, and BCI experiments to filter out these common movements, and used the recorded EEG data to determine the IAFs using curve fitting methods.

By checking the effect of online artefact removal on peak detection, we can grasp how effective is it to measure attention and fatigue-related EEG parameters while the users are mobile. For BCIs whose target user group is healthy people, mobility or movements are expected and indeed necessary to account for. Although the exact parameters or combination of them for describing objective user fatigue or attention is not clear yet, IAFs can be used as a base for many parameter estimations. Once determined, the negative effects of fatigue could be countered by altering BCI parameters. For both artefact removal and peak detection we utilised novel methods and algorithms. In our study we used IAF measurement methods as an indicator, or starting step for determining objective fatigue. However, it is out of the scope of this paper to make measurements or assumptions measuring either fatigue or attention during BCI use. Additionally we measured the effect of the artefact removal on BCI performance. However, the main goal remains the evaluation of the artefact removal combined with peak detection during BCI use. Even if BCI performance is worse using these methods, it can be easily amended by simply not filtering the data for BCI classification.

First, the materials are described, followed by the description of the implementation and changes to the artefact removal method, followed by the peak detection method and finally the experimental protocol including the used BCI system. The results of the simple EEG recordings and the BCI use are detailed separately and discussed in the respective sections of the paper.

## 2. Materials and Methods

### 2.1. Participants

Fifteen healthy users (eight female) participated. All of them were students of the Rhine-Waal University of Applied Sciences. The average age (SD) was 23.8 (2.9) years. After discussing the experimental protocol with each subject, they signed a consent form according to the Declaration of Helsinki. The experiment was approved by the ethical committee of the Medical Faculty of the University Duisburg-Essen. Subjects were paid a small fee to participate in the experiment.

### 2.2. Hardware

For recording EEG, a biosignal amplifier, g.USBamp (Guger Technologies, Graz, Austria) was utilised. Passive Ag/AgCl electrodes were used to measure EEG signals, fourteen of them were placed at the following positions: P${}_{Z}$, PO${}_{3}$, PO${}_{4}$, O${}_{1}$, PO${}_{Z}$, O${}_{2}$, O${}_{9}$, O${}_{10}$, P${}_{3}$, C${}_{Z}$, P${}_{4}$, F${}_{1}$, F${}_{Z}$, and F${}_{2}$ according to the international 10-5 electrode placement system. AF${}_{Z}$ was used as a ground, and the left earlobe was used for reference. Before the experiment, all electrodes were prepared with an electrolytic gel to lower impedances below 5 kΩ.

Data were recorded at a sampling rate of 256 Hz. A bandpass filter between 2 Hz and 60 Hz, as well as a notch filter between 45 Hz and 55 Hz to remove power line noise were applied. These filters were applied before utilising the artefact removal method, specified in Section 2.4. Data were sent to be processed in blocks of 64 samples (250 ms long recordings).

The BCI program, including the graphical user interface (GUI) and all calculations, ran on a laptop (MSI GE72MVR 7RG Apache Pro (17.3 inches), resolution: 1920 × 1080 pixels, vertical refresh rate: 120 Hz, Intel(R) Core(TM) i7-7700HQ CPU @ 2.80 GHz, operating on Microsoft Windows 10 Education).

### 2.3. Experimental Protocol

The experiment was divided into three phases, each with its subsequent tasks. After signing the consent form, subjects were prepared for recording. Electrode gel was applied at all recording sites. This was followed by the first phase, setting up the artefact removal filter (Section 2.4).

In the filter setup phase users had to follow the instructions on screen, altogether six of them, while keeping their gaze as much as possible in the middle of the screen, marked by a cross. Between the tasks they could take short breaks as needed, during which no EEG data were saved. Each task required a specific activity for ten s, and they were the following: blink, move your head, do face movements/gesticulation and remain as still as possible (for three times in total). The order of the tasks was randomised. The data recorded during these tasks was used to create the multi-channel Wiener filter. For further details on this phase please see Section 2.4.

After the filter has been created, in the second phase of the experiment, users had to do ten specific tasks. The tasks were in a randomised order, presented by the GUI. During this phase there was no stimulation presented to the users; simply EEG was recorded. The tasks were the combination of three distinct settings, eyes open or closed, filter on or off, and relaxing/imagining movements/doing movements. The relaxing conditions with both eyes open and closed were only measured without the filter being on, as there was nothing to filter out and consequently precise results were expected even with less data, thus resulting in 2 × 2 × 3 − 2 = 10 tasks. The GUI did not show any information about the filter, only whether to keep eyes closed/open and what activity to do. Subjects were instructed to do any combination of the movements they had done during the first phase, but not any new type of movement. Each user could freely select any combination of the movements done previously, however during the recording they were monitored by the experimenter to assure they kept moving/still during the whole recording as per instructed. If not all conditions were fulfilled (e.g., a subject opened his or her eyes, did not keep still, or did not move according to the specific task) the task was repeated. Each task had to be maintained for 45 s, during which EEG was recorded.

For the third phase of the experiment, users had to use an SSVEP-based BCI speller to write a word selected by them (approximately 5–6 characters long, or 15–18 selections with the BCI, respectively, but otherwise not specified). Due to its robustness in previous experiments, a three step speller was used (see Section 2.5.2). The same word had to be written thrice while executing one of the tasks: keeping as still as possible, moving according to the movements in phase one of the experiment, and the same movements, but with the filter active. For the keeping still task, no filter was used. However, during the later offline analysis the filtered data was calculated from the corresponding unfiltered data. The state of the filter was, again, not shown to users, and they were instructed when necessary by the experimenter not to stop moving, similarly as in phase two. These three tasks were randomised as well. The experiment was finished if the user has written the preferred word thrice, or if after a prolonged time no progress was made towards finishing the spelling task. In the latter case the condition was deemed unsuccessful for spelling with the BCI.

**Figure 1:**
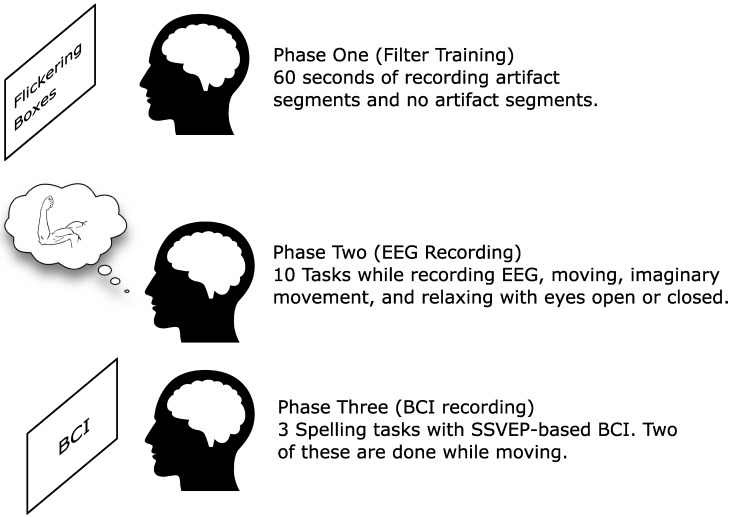
Three phases of the online experiment. Electroencephalography (EEG), brain–computer interface (BCI), steady-state visual evoked potential (SSVEP).

The whole experiment lasted approximately one hour, with possibilities for short breaks between the tasks (when needed by the users) For an overview of the phases of the experiment, please see Figure 1.

### 2.4. Artefact Removal

The artefact removal algorithm presented by [21] was adopted for this experiment. As a summary of their method: The algorithm calculates an estimate of the multi-channel artefact signal by linearly combining the channels of observations. The input data, as mentioned before, is labelled as artefact segments and artefact-free segments, which are used in acquiring an optimal filter solution using only covariance matrix estimates. The neural responses can be obtained by subtracting this estimated artefact from the data.

Furthermore the spectral properties of the data (different effects on different electrode locations) are also considered, and the algorithm can be extended by performing a finite impulse response filtering in each channel, which acts as a per-channel spectral filter. This way, the algorithm can optimally remove the pre-defined artefact types while at the same time minimises the removal of actual EEG components. For a detailed description of methods and calculation please refer to [21].

This algorithm was applied in our custom made BCI program (Section 2.5.2), which was also handling EEG recordings. For this, the algorithm was made automatic and online, calculations being done after each block of recorded EEG data (every 250 ms) to remove possible artefacts before being processed by the BCI classification. For the automation a training phase was added, where the generic marking of artefact segments was done.

The experiment started with this training phase for creating the filter. Users were asked to follow instructions presented in the GUI, which told them to remain as still as possible for several seconds or to do movements. Six tasks were listed in a randomised order, requiring blinking, head movements, face movements/gesticulation and remaining as still as possible (three times in total), for ten seconds each. The instructions were closely monitored by the experimenter, and if any deviation from them was noted, the whole phase was repeated. There were no limitations on the movement types, e.g., for head movements nodding or shaking of the head could be done as well as any other head movement, however subjects were instructed to remember the movements they made in this phase and replicate them in the later phases. By specifying whole data segments of this phase as artefact segments or clean segments, the manual marking was omitted, making the artefact removal automatic. This, however, could have influenced the performance of the removal process. In a practical application the users are not in the presence of experts, and since their knowledge of EEG artefacts can be minimal or even non-existent, they cannot be asked to mark these artefacts segments themselves.

During this training phase, four targets were flickering, precisely as in the spelling task later, with the same frequencies. This was done to mitigate the effect of the filter on the signal the BCI was searching for. Although there was flickering, no interaction was possible for the users (no selection, no feedback, no classification). Users were instructed to do the movements while looking at the middle of the screen, marked by a cross. Thus the four flickering targets were in their visual field, but the users were not looking directly at them. The tasks where users had to remain still were marked as artefact-free data, while all other recordings were marked as artefacts.

The filter was created with a delay of four samples. This means that time-lagged versions of each channel (up to ±four samples, or ∼16 ms) are stacked to the observation matrix, which is used to calculate the multichannel Wiener filter. This delay was selected considering the temporal and spatial effects (e.g., blink artefacts can have large autocorrelation coefficients after tens of milliseconds) of artefacts on EEG. For details please refer to [21]. The resulting filter was a 126 × 126 matrix, 9 (from −4 to +4 sample time lags) × 14 (electrodes). This was applied to each incoming block (14 electrodes × 64 samples) of data when the filter was active. First, the time-lagged versions of the incoming data were calculated with zero-padding to match the original dimensions and the dimension of the filter. Then the artefact estimate was calculated for the original (not time-lagged) channels using the filter. The artefact estimate was then removed from the EEG to get the filtered data as described in [21].

### 2.5. Offline Analysis

After the experiment, an offline analysis was conducted to determine peak frequencies in the theta, alpha, and beta ranges. The program recorded EEG data after the notch filtering at 50 Hz and the bandpass filtering between 2 Hz and 60 Hz in every case (referred to as unfiltered from here on), and additionally when the artefact removal filter was operational, it recorded the data after the removal process as well (referred to as filtered from here on). The offline analysis was done differently for the data recorded in phases two and three of the experiment.

In phase two, after the training phase for the artefact removal, the users had to do different movement-related tasks or imaginary movements while EEG data were being recorded. In the cases where the artefact removal was not applied online during the recording, the filtered data was calculated offline from the corresponding unfiltered data, this way providing more data for the statistical analysis, providing more robust results. Altogether ten different tasks were required from the users, and for both the unfiltered and the filtered data the peak detection algorithm from [11] was applied to find IAFs.

First, the FFT was normalised, then a smoothing filter (Savitzky–Golay filter) was applied. Potential alpha peaks were determined by finding zero crossings in the first derivative of the smoothed normalised FFT. If there were multiple crossings, the highest peak was used. If the peak was higher than the next highest peak by a predefined threshold, it became a peak frequency. Inflexion points were determined from the second derivative of the FFT, and the area under the curve (*Q*) between the two inflexion points around the peak was calculated.

The EEG data were separated into three sets, according to the spatial position of the recording electrodes. The occipital area had seven recording electrodes: PO${}_{3}$, PO${}_{4}$, O${}_{1}$, PO${}_{Z}$, O${}_{2}$, O${}_{9}$, and O${}_{10}$ the centro-parietal area consisted of P${}_{Z}$, PO${}_{3}$, PO${}_{4}$, P${}_{3}$, C${}_{Z}$, and P${}_{4}$, while the frontal area encompassed only F${}_{1}$, F${}_{Z}$, and F${}_{2}$. This was done, so the results are easier to present here.

#### 2.5.1. Center of Gravity

Center of Gravity (CoG) calculation is a more global method of peak frequency detection, as information is given about the shape of the peak as well, and even not so pronounced peaks can be detected. First, the FFT of the epoch has to be calculated, and a frequency range specified for the calculations. Originally, as proposed by Klimesh et al. [22], this was done by visually inspecting the signal, and finding the beginning of the “ascent” and the end of the “descent” of the alpha peak. The peak frequency is calculated using the following formula:

(1) IAF=∑(a(f)xf)/∑a(f),

where IAF is the individual peak frequency, and a(f) is the power spectral estimate from the FFT at frequency *f*. The frequency range for this equation was the previously determined start of “ascent” and end of “descent”.

Corcoran et al. [11], used the centre of gravity calculation with an automatic search for the frequency range. After finding the alpha peak as described above, for the CoG calculation the first derivative of the FFT was searched for local minima or near horizontal functions prior and after the peak. If there were multiple minima before/after the peak, the closest ones to the peak were used.

#### 2.5.2. BCI

Classification was done only during the third phase of the experiment when the users had to write a word using BCI. In the other phases the EEG data was only processed using the method described in Section 2.4 and offline.

Minimum energy combination (MEC) was used to classify the recorded EEG data, similarly to [23]. MEC combines the data from the least noisy channels. The amount of noise in the data recorded by each channel is calculated by removing the target signals and assessing the rest. The combination is executed for each target frequency, resulting in comparable SNR measures for all targets. After normalization, the result of the classifier is given in percentages for each investigated frequency. For more details please see [23].

In our case, not a single classifier, but four of them were analysing data simultaneously. The only difference between them was the amount of data to analyse, which was 3, 4, 5, and 6 s respectively. The calculations of a classifier started only if enough data were available (for example the 5-second classifier started after 5 s of recording). However, once started they would analyse after each block of incoming data, by shuffling out the oldest block of data and appending the new block. If more than six seconds of recording were available, all four classifiers were calculating simultaneously. The results were combined by weighted averaging, by assigning the highest weights to the longest classifiers. For more details please see [24].

After selecting a target, the flickering stopped for two seconds, and the data recording for the classifiers was started from the beginning (3, 4, 5, 6, or more seconds were needed again for the respective classifiers to start calculating). This gaze shifting time was implemented to allow users to find the next target and to allow the dissipation of the SSVEPs generated by the previous flickering.

In phase four flickering targets were utilised, however, to reduce the occurrence of false positives, three additional frequencies were involved in the calculations of the classifier, without being presented to the users as flickering stimuli. If one of the additional stimuli was classified, no output was produced. The four target frequencies were: 14.0 Hz,14.2 Hz,14.4 Hz, and 14.6 Hz, while the additional three (not displayed) were: 14.13 Hz,14.33 Hz, and 14.53 Hz. This makes measuring activities in the different wavebands easier as there is minimal overlap. The range 14–15 Hz is above the traditional alpha range and the expected peak alpha frequency. Conversely, the range is below the expected peak beta frequency and as such the SSVEPs should have little or no effect on finding these peak values.

#### 2.5.3. Graphical User Interface

The GUI was presented to the users throughout the entire experiment. However, the users could not interact with it during the first two phases. No selection was possible there, and in phase two even the flickering effect was not present. For the third phase (BCI spelling task) it had full functionality.

**Figure 2:**
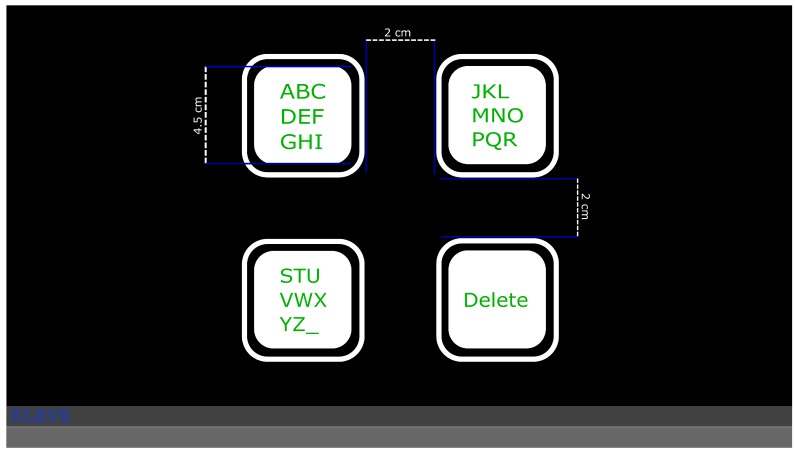
An image of the GUI, with the size of the stimuli and the distances between them. Users were looking at it from approximately 80 cm distance. The stimuli are the smaller squares, surrounded by non-flickering frames, and the length of their sides are equal.

This speller was a three-step speller; three selections were necessary to write any letter, similar to [24]. Twenty-seven characters (letters of the English alphabet and ’_’ for space) were organised into three flickering targets in the initial layout, and an additional target enabled the users to delete the last written character (Figure 2). Each selection narrowed down the presented characters to the ones contained in the selected target, e.g., selecting the first target from the initial layout resulted in the first nine characters of the English alphabet to be distributed among three targets. If a target was selected that only contained a single letter, that letter was written. At each layout other than the initial, the option to go back to the previous layout was provided to the users (replacing the delete option). For more details on the spelling logic please see for example [25].

## 3. Results

### 3.1. Peak Detection

With the methods mentioned in Section 2.5, an alpha peak was determined for each subject and each electrode with each task. To provide more compact, comprehensible results, the electrodes have been grouped according to their areas into three groups, occipital, centro-parietal, and frontal. Between the first two groups there was a small overlap, PO${}_{3}$, and PO${}_{4}$ are a part of both groups.

The parameters for the offline analysis, according to [11] were as described above (e.g., the sampling rate was 256 Hz, 45 s of EEG data was used for 14 recording electrodes). Additionally, alpha peaks were expected in the range of 7–14 Hz, with the Savitzky–Golay filter of frame width 15 and ninth polynomial order. In the case of competing peaks 20% peak height difference was set as the threshold, and the minimum number of channel estimates to resolve for calculating average CoG and peak alpha frequency estimates were 4, 3, and 2 for the occipital, centro-parietal, and frontal areas, respectively.

**Figure 3:**
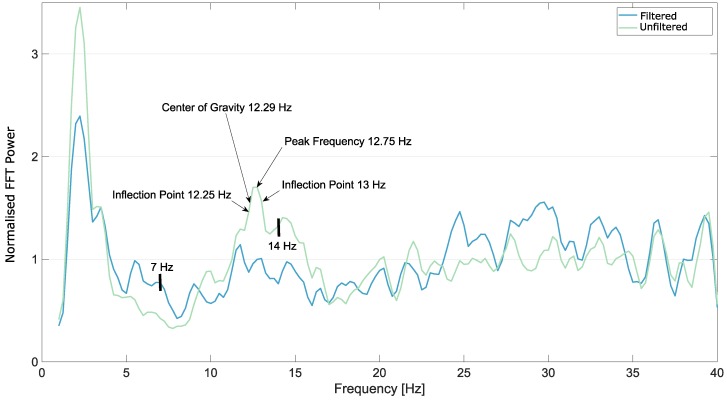
Smoothed fast Fourier transform (FFT) from Subject 1, electrode O${}_{Z}$, eyes open, executing movements. The artefact removal in this case did not result in better peak detection. The borders of the automatic detection range, 7 Hz and 14 Hz are marked on the plot as well.

**Figure 4:**
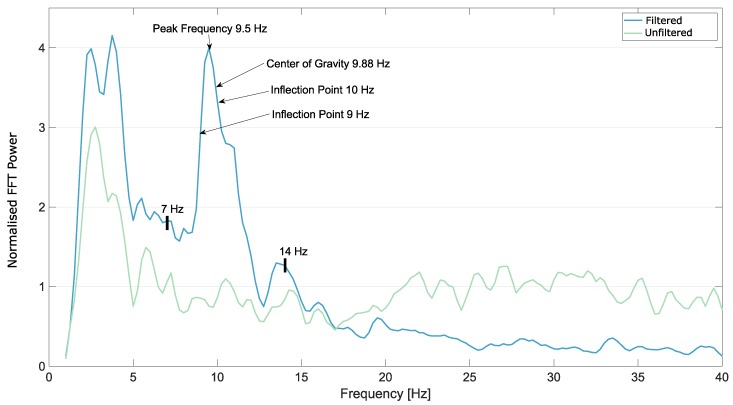
Smoothed FFT from Subject 3, electrode O${}_{Z}$, eyes open, relaxing. The artefact removal in this case resulted in better peak detection. The borders of the automatic detection range, 7 Hz and 14 Hz are marked on the plot as well.

**Figure 5:**
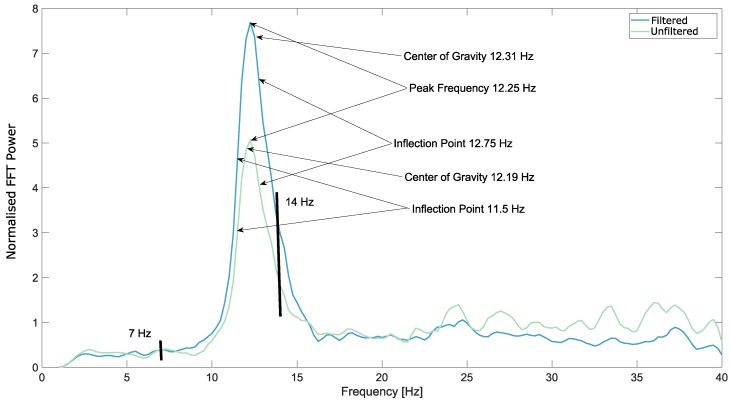
Smoothed FFT from Subject 5, electrode O${}_{Z}$, eyes open, imagining movements. In this case, both the unfiltered and the filtered data could be used to find a clear peak. The borders of the automatic detection range, 7 Hz and 14 Hz are marked on the plot as well.

A few examples of the FFT from electrode O${}_{Z}$ from the occipital area, after the Savitzky–Golay smoothing filter was applied, are shown in Figure 3, Figure 4 and Figure 5.

For each area specified, a mean and standard deviation were calculated. Peaks of individual channel estimates were weighted by $Q_{f}$, which is calculated by dividing *Q* (area under the curve between the inflexion points) by the bandwidth of this range (the number of frequency bins between the inflexion points). $Q_{f}$ aims to quantify the relative strength of each channel peak, as described by [11]. As an example, the results of Subject 6 are shown in Table 1.

The measured peaks were manually inspected to check the results provided by the algorithm. This was done for each electrode separately. The previously used areas are not used here. The results of the manual inspection are shown in Table 2.

**Figure 6:**
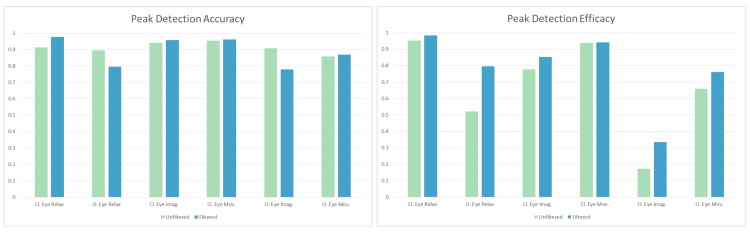
The accuracy and efficacy (true positive divided by the total) of the second phase of the experiment, shown individually for each task the users had to do during the recordings. “Cl.” notes tasks with closed eyes, “O.” is for open eyes, “Imag.” notes imaginary movement, and "Mov." stands for actual movements. The results of the same task from the online recordings were averaged, e.g., “Cl. Eye Mov.” shows the combined accuracy and efficacy of two tasks. As mentioned in Section 2.3 the relaxation tasks were only done once, every other case contains twice the amount of data.

Using this confusion matrix, the accuracy of the automatic peak measurement can be assessed by dividing the number of true negative and true positive cases by the number of all cases. An online tool for accuracy calculation can be found at https://bci-lab.hochschule-rhein-waal.de/en/acc.html. It was found to be 89.69% accurate if manual inspection is considered ground truth. The true correct peaks divided by all cases can be used to compare the efficacy of the algorithm in finding peaks in different scenarios. The comparisons with these values are solely used here for grasping the difficulty of finding alpha peaks in different scenarios. The tasks were inspected individually, each with its confusion matrix, and the summary of these results is shown in Figure 6, with the accuracy and the previously mentioned efficacy.

The accuracy results from Figure 6 are mixed; in some cases the unfiltered data is more accurate; in other cases the artefact removal increases accuracy. The average difference is 2.18%, with the unfiltered data being more accurate. However, the efficacy, or how often a true peak was found in the different tasks, is always higher for the filtered data (Figure 6). In some cases there is not much difference, e.g., with closed eyes relaxing there is only a 3.22% increase, but in the most pronounced case, with eyes open and relaxing, 27.51% more alpha peaks were found when the artefact removal was applied.

To statistically evaluate the effectiveness of peak detection with and without artefact removal, the cases when the peak was successfully determined were compared. For each subject, each electrode and each task the results of the peak detection were deemed unsuccessful if no peak could be determined with the above-mentioned settings, or successful if a peak was found. This way 2100 (15 Subjects, 14 Electrodes, and 10 Tasks) data points were gathered for both the filtered and unfiltered conditions. The comparison was done by using McNemar’s test, for both the automatic detection method (without changing false positive and negative findings) and the manually corrected results. The automatic results showed a significant difference (p<0.0001), with an average successful detection rate of 65% for the unfiltered condition and 72% for the filtered condition, while the manual results also showed a significant difference (p<0.0001), with detection rates of 67% and 78% respectively. By examining the tasks separately (merging data from separate recordings with the same task), McNemar tests show significant differences for the eyes closed relaxing task, p=0.011 and p=0.004 for the automatic and manual results respectively and for eyes closed moving, eyes open relaxing, eyes open imaginary movement, and eyes open moving conditions, p<0.0001 in every case for both automatic and manual results. The only not significantly different condition was with eyes closed and imaginary movement, p=0.70 and p=0.286 for the automatic and manual results respectively. For all cases which were significantly different, the filtered data provided a better rate of finding peaks.

The results were also compared task-wise to see if different alpha peaks were detected using the filtered/unfiltered data. For this comparison, the grouped data was used, with a separate condition for each group. For the occipital group at least four out of the seven electrodes needed a determined peak to calculate an average, for the centro-parietal area this was three out of six, and for the frontal area, two out of three. As in some cases no peak was detected, the number of samples for which this comparison could be done was varied and limited. The measured average values were compared for each task separately with a repeated measures two factor ANOVA, as this enabled more samples for some of the comparisons, and the data were checked with regard to filtering (movement artefacts filtered out in the specified way or not), and the peak detection method (curve fitting or center of gravity). The results are shown in Table 3.

As can be seen from the table, the majority of the detected peaks were not significantly different. More details about the significant differences are shown in Table 4. The largest difference in means when the filtering is concerned is 0.141 Hz. Regarding the selected algorithm, the difference of means can be as high as 0.353 Hz. If more precise peak detection is necessary in the future, the parameters of the artefact removal and peak detection methods likely have to be adjusted.

### 3.2. Peak Detection During BCI Phase

The analysis of IAF during BCI use was done as it would be for fatigue or attention level analysis. This means the EEG data was separated into flicker-free and flickering segments. These segments were then further subdivided into different time segments of the recording. This way, peak detection could be done for different time segments of the experiment, which could be used to track the properties of the IAF throughout the recording. For all these segments the manual inspections proved to be more complicated than the previous phase due to the frequent lack of clear peaks, and even when peaks were found they were mostly flat or distorted. The analysis of the flicker-free and flickering segments was done on an electrode basis (not grouped into areas) and is discussed separately in the next sections.

#### 3.2.1. Flicker-Free Segments

**Figure 7:**
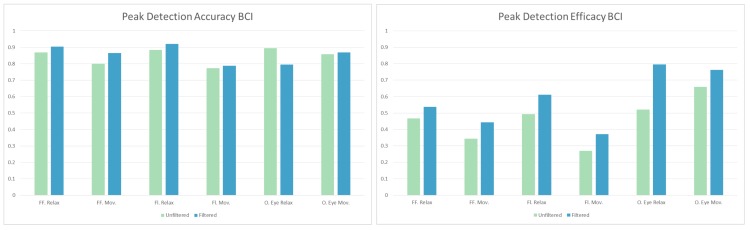
The accuracy and efficacy (true positive divided by the total) of the third phase of the experiment, shown for using the BCI while relaxing/moving, as well as open eyes while relaxing/moving from phase two.

After every selection, a two-second gaze shifting time was implemented in the BCI, for the users to have enough time to find the next target letter. During this gaze shifting time the flickering of the stimuli was turned off. These segments were merged and divided into eight-second long segments (or 4096 samples with the sampling rate 256 Hz). These segments were separately checked for peaks in the alpha range with the curve fitting method described above. This was followed by manual inspection, and the results of this are shown in Table 5. The accuracy of the automatic detection is 85.12% if the manually detected peaks are considered ground truth. The accuracy and efficacy (as described above) values calculated for the two cases are shown in Figure 7, together with the same results from the flickering segments, which are described in the next section.

#### 3.2.2. Flickering Segments

The rest of the recordings during BCI use (when the stimuli were flickering), were merged and divided into 15 second long segments for this analysis. Since the recording time was different for each subject, the available data was also varied. The length of the segments (15 s) was chosen to provide at least three segments from each participants’ data. For this analysis, all subjects’ EEG data was used, regardless of whether they could finish the BCI spelling task or not. The peak detection method was slightly altered to avoid mistakes from the SSVEPs. The boundaries for the search were altered, to 8–13 Hz (this way the ∼14 Hz SSVEP and ∼7 Hz subharmonic were both excluded from the range). The following manual inspection results are shown in Table 6 and Figure 7.

### 3.3. BCI Performance

The accuracy, the total time and the Information Transfer Rate (ITR) of the spelling tasks were used to evaluate the BCI performance of the three different scenarios, relaxing, moving without applying artefact removal, and moving while applying artefact removal. An online tool for ITR calculation can be found at https://bci-lab.hochschule-rhein-waal.de/en/itr.html. These measures are shown in Table 7 together with the mean and SD for the scenarios. The cases when users could not finish the spelling tasks are excluded, and not used in the statistical evaluation. The scenarios were compared with one factor repeated measures ANOVA, and the respective results are p=0.036, p=0.001 and p=0.011 (with Greenhouse-Geisser correction) for the accuracy, ITR and spelling time respectively. All three parameters show significant difference.

Further investigations were done by pairwise tests (paired t-tests). For brevity, the relaxing scenario is marked RE, the moving without filter MW, and the moving while filtering MF, from here on. The pairwise accuracy results are: p=0.556, p=0.024, and p=0.085 for RE-MW, RE-MF, and MW-MF, respectively. For ITR: p=0.048, p=0.003, and p=0.014 for RE-MW, RE-MF, and MW-MF, respectively. Finally, for spelling time: p=0.020, p=0.012, and p=0.049 for RE-MW, RE-MF, and MW-MF, respectively. This translates into the MW and MF scenarios performing significantly worse than the movement-free scenario in every aspect, except accuracy, which is not different significantly between the MW and RE scenarios.

Additionally two subjects could not finish the spelling task with the MW scenario, and one could not finish the task with the MF scenario. Furthermore, the MF scenarios’ performance is worse than the MW scenarios’ regarding ITR and spelling time. The potential causes of this are examined in the Discussion section.

## 4. Discussion

The online automatic artefact removal method applied proved to improve IAF detection. For simple EEG recordings the method from [21] proved to result in data significantly better for determining alpha peaks using the described curve fitting method from [11]. The detection rate increased from 64.87% to 71.52%. After manual inspection and correction the increase is from 66.57% to 78.48%. The increase was even more pronounced in some cases, especially while the users were relaxing with open eyes.

Generally, movements did not affect alpha peak detection, which is likely the combined result of several effects. Movement control is associated with the central brain area, which in this experiment was measured by a single electrode. The effect of these potentials in the central area did not influence the occipital area, where most recording electrodes were located. Furthermore the movements did not require any visualisation or precision, and thus while the movements were executed by the users likely the visual cortex pertained a high alpha activity, possibly even higher than in a relaxing condition.

As opposed to movement-related artefacts not influencing the alpha peak detection, imaginary movement tasks resulted in a more considerable decrease in the amount of found peaks. Without artefact removal, this decrease was 17.61% and 35.00% of the total for the closed and open-eyed conditions, respectively. With artefact removal it changed to 13.23% and 46.18% of the total, respectively. This effect is likely the result of users not trained for executing imaginary movement tasks; thus they were likely visualising the movements. This, in turn, decreases alpha activity in the occipital area, and a peak is harder to detect. In these cases the artefact removal algorithm made the peak detection substantially more effective.

The most substantial effect of filtering, however, was for the eyes open and relaxing condition. The reason for this is hard to pinpoint and requires further investigation. One reason could be that users were inspecting their surroundings during this task, which would then reduce alpha activity in the occipital area.

When the SSVEP-based BCI was used by subjects while relaxing, compared to the open eyes relaxing condition from phase two, there was no substantial decrease in efficacy (5.33% and 2.84% of the total for the flicker-free and the flickering conditions, respectively), when checking the unfiltered results. With artefact removal, the differences increased to 25.96% and 18.61% of the total. These differences confirm the degradation of peak detection efficacy when our SSVEP-based BCI is used. The substantial change in efficacy after artefact removal is due to the substantial improvement of peak detection in phase two for the eyes open and relaxing condition.

When users were moving during BCI use, the decrease was 31.61% and 38.94% of the total for the flicker-free and the flickering conditions with the unfiltered data, respectively. With artefact removal, these differences remained high, 38.94% and 39.05% of the total, respectively. This result shows that BCI use while the users are moving effects the detectable peaks substantially.

Although the BCI use, especially with the users moving degraded peak detection by a large amount, the artefact removal provided an improvement in every condition, the efficacy (true positive rate) increased on average by 9.68% of the total (combining flicker-free and flickering conditions, as well as relaxing and moving scenarios) if the filtering is applied.

The performance of the BCI decreased for the scenarios when users were moving as expected. However, the filtering had no positive effect. Instead the opposite occurred, performance was worse than without filtering. There can be several reasons for this, the most obvious one being the filter affecting the prevalence of the SSVEPs generated by the flickering. This could be the result of setting up the filter, and defining the coefficients for filtering. If the SSVEP during this phase (phase one of this experiment) is not comparable in strength to the one during the experiment, the filter can decrease the power of the frequency component during the online task to the levels of the training phase, which decreases BCI performance. As shown by [26], attention affects the properties of the generated activity, and, as in our experiment, users were instructed to look at the middle of the screen in the training phase, not directly at the stimuli, the decrease in performance can very well be a consequence. To investigate this and other potential causes, further studies are necessary.

Another important detail of note is the classification method, MEC, which selects the least noise-ridden electrodes data for classification. As in our case, eight electrodes were providing data for the classifier; if half of them were influenced by artefacts, the four remaining could still be used to classify without too much difficulty. This can be another factor in the performance difference between the filtered and unfiltered BCI tasks. As the classification method is robust against noise, and the artefacts in this experiment did not cause enough noise to necessitate the artefact removal, the only effect of the filter was the attenuation of SSVEP responses, which resulted in slower classification. The use of fewer electrodes to necessitate the use of slightly noisy electrode channels or the use of a different classification algorithm can be used to investigate this effect further.

Although the detection rate can be improved, during BCI use a considerably lower number of peaks was found. This can be the result of a less boring task (BCI use), or change in mental fatigue, or just from the difficulty of finding peaks during BCI use. However, the investigation of this is not in the scope of this paper. Peak alpha frequency determined during the eyes open condition for example can be measured with the filtering method (providing an improvement), which could then be used to calculate the individual alpha range and provide a list of other parameters based on the alpha range (area under the curve, power in the lower alpha range/higher alpha range etc.).

## 5. Conclusions

The measurement of objective user fatigue can provide a way to minimize its negative effects on BCI performance, by adjusting parameters of the BCI accordingly. Methods which help to measure user fatigue objectively, accurately, and online are therefore highly beneficial for BCIs that are planned to be used for a prolonged time. As mentioned previously, BCIs in the future are expected to be used in everyday, practical scenarios. In these cases, movements can cause artefacts in EEG recordings, this was also observed in this experiment as well. Therefore, some movements have to be anticipated and handled accordingly. The measurement of fatigue or attention with EEG has the same difficulties regarding movement artefacts, as the cases where fatigue measurements are needed are always related to some activity, e.g., after prolonged driving phases. The presented online automatic artefact removal showed an average 9.68% of the total increase in the number of peaks found during an SSVEP-based BCI use, where the users were moving to generate artefacts. When no BCI was used, the increase was on average 10.91% of the total.

This means that the used techniques are beneficial for detecting the peak alpha frequency from the EEG recordings, even during BCI use under noisy conditions. Utilising artefact removal and curve fitting for peak alpha frequency measurements, the basis for determining objective fatigue accurately was greatly improved. These results were achieved without restrictions on the specific movements. Furthermore, the artefact removal was trained in a generic way without a specific artefact detection algorithm. Refining the parameters of both the removal method and the peak detection algorithm can lead to even better results, as can the implementation of an online artefact detection algorithm.

Our experiment employed movements which would occur in everyday scenarios, such as talking or chewing gum. The next step regarding mobility will be testing in a noisy environment using a mobile amplifier.

Regarding objective fatigue and attention measurement, increasing the number of measured parameters is planned, e.g., by determining theta peaks, extracting phase information for alpha peaks, as well as assessing the spatial information, to find a reliable way to measure fatigue and at the final end e.g., the level of attention of BCI users.

To conclude, the presented system provided, on average, a nearly 10% (of the total) increase in the amount of detected alpha peak frequencies. Even when users were moving or executing imaginary movement tasks it provided a better peak finding rate. With small adjustments, further improvements can be expected, providing a promising way towards extracting information from EEG in practical, everyday, mobile scenarios.

## Figures and Tables

**Table 1:** Examples of the calculated peak alpha frequencies with the curve fitting and Center of Gravity (CoG) methods for Subject 6. The results are presented by task, with “Mov.” indicating movements during the recording and “Imag.” indicating imaginary movements done by the subject. The column “Ch.” lists the number of channels that were used to calculate the Peak and SD values. If a channel could not be used for detecting a peak, it was excluded from the average calculation. For the Occipital, Centro-Parietal and Frontal areas 4, 3, and 2 electrodes were the minimum to calculate the average peaks, respectively.

		**Occipital**											
		**Curve Fitting**						**Center of Gravity**					
**Eyes**	**State**	**Unfiltered**			**Filtered**			**Unfiltered**			**Filtered**		
		**Peak**	**SD**	**Ch.**	**Peak**	**SD**	**Ch.**	**Peak**	**SD**	**Ch.**	**Peak**	**SD**	**Ch.**
		**(Hz)**			**(Hz)**			**(Hz)**			**(Hz)**		
**Closed**	**Relax**	9.43	0.12	7	9.47	0.09	7	9.48	0.02	7	9.51	0.04	7
**Open**	**Relax**	10.80	0.17	7	10.79	0.09	7	10.66	0.05	7	10.70	0.03	7
**Closed**	**Mov.**	9.88	0.13	7	9.96	0.09	7	9.77	0.03	7	10.04	0.03	7
**Closed**	**Mov.**	9.75	0.00	7	9.78	0.09	7	9.74	0.05	7	9.81	0.03	7
**Closed**	**Imag.**	9.53	0.09	7	9.71	0.09	7	9.67	0.04	7	9.84	0.04	7
**Closed**	**Imag.**	9.64	0.13	7	9.75	0.00	7	9.69	0.03	7	9.76	0.02	7
**Open**	**Mov.**	-	-	0	-	-	0	-	-	0	-	-	1
**Open**	**Mov.**	-	-	3	-	-	0	-	-	3	-	-	0
**Open**	**Imag.**	10.50	0.00	7	10.50	0.00	7	9.85	0.06	7	10.05	0.02	7
**Open**	**Imag.**	10.50	0.00	7	10.50	0.00	7	10.11	0.06	7	10.25	0.01	7
		**Centro-Parietal**											
**Eyes**	**State**	**Curve Fitting**						**Center of Gravity**					
		**Unfiltered**			**Filtered**			**Unfiltered**			**Filtered**		
		**Peak**	**SD**	**Ch.**	**Peak**	**SD**	**Ch.**	**Peak**	**SD**	**Ch.**	**Peak**	**SD**	**Ch.**
		**(Hz)**			**(Hz)**			**(Hz)**			**(Hz)**		
**Closed**	**Relax**	9.50	0.00	6	9.50	0.00	6	9.50	0.04	6	9.57	0.03	6
**Open**	**Relax**	10.85	0.13	6	10.85	0.13	6	10.70	0.08	6	10.67	0.06	6
**Closed**	**Mov.**	9.71	0.10	6	9.82	0.13	6	9.76	0.06	6	10.05	0.08	6
**Closed**	**Mov.**	9.75	0.00	6	9.87	0.14	6	9.83	0.05	6	9.84	0.03	6
**Closed**	**Imag.**	9.70	0.10	6	9.75	0.00	6	9.78	0.06	6	9.90	0.02	6
**Closed**	**Imag.**	9.67	0.13	6	9.75	0.00	6	9.71	0.05	6	9.72	0.04	6
**Open**	**Mov.**	-	-	0	-	-	0	-	-	0	-	-	2
**Open**	**Mov.**	-	-	0	-	-	0	-	-	0	-	-	0
**Open**	**Imag.**	10.52	0.16	6	10.46	0.10	6	9.69	0.18	6	9.93	0.07	6
**Open**	**Imag.**	10.40	0.21	6	10.39	0.21	6	10.14	0.14	6	10.21	0.08	6
		**Frontal**											
		**Curve Fitting**						**Center of Gravity**					
		**Unfiltered**			**Filtered**			**Unfiltered**			**Filtered**		
		**Peak**	**SD**	**Ch.**	**Peak**	**SD**	**Ch.**	**Peak**	**SD**	**Ch.**	**Peak**	**SD**	**Ch.**
		**(Hz)**			**(Hz)**			**(Hz)**			**(Hz)**		
**Closed**	**Relax**	9.25	0.00	3	9.50	0.00	3	9.37	0.01	3	9.47	0.01	3
**Open**	**Relax**	10.50	0.00	3	10.75	0.00	3	9.24	0.03	3	10.06	0.01	3
**Closed**	**Mov.**	9.75	0.00	3	9.75	0.00	3	9.59	0.01	3	9.78	0.00	3
**Closed**	**Mov.**	9.75	0.00	3	9.75	0.00	3	9.77	0.00	3	9.83	0.01	3
**Closed**	**Imag.**	9.50	0.00	3	9.75	0.00	3	9.70	0.01	3	9.69	0.00	3
**Closed**	**Imag.**	9.50	0.00	3	9.75	0.00	3	9.66	0.00	3	9.59	0.01	3
**Open**	**Mov.**	-	-	0	-	-	0	-	-	0	-	-	0
**Open**	**Mov.**	-	-	0	-	-	0	-	-	0	-	-	0
**Open**	**Imag.**	10.58	0.14	3	10.00	0.00	3	9.47	0.01	3	9.61	0.01	3
**Open**	**Imag.**	10.42	0.14	3	10.00	0.00	3	9.97	0.01	3	9.89	0.00	3

**Table 2:** Manual inspection of peaks from phase two of the experiment. Values in parenthesis mark cases when the automatic and manual inspections both found peaks, however the peaks were more than 0.5 Hz apart, so they are considered wrong. The not parenthesized numbers in the same cells include these cases.

Manual Inspection		Manual Peaks			
		Unfiltered		Filtered	
		Peak Found	No Peak	Peak Found	No Peak
**Automatic Peaks**	**Peak Found**	1266	70 (10)	1447	41 (6)
	**No Peak**	113	651	189	423

**Table 3:** The results of the comparison (repeated measures two factor ANOVA) of the detected peaks for all tasks. The values are *p* values, the significant results (under 0.05) are marked with an ‘*’. Methods are CoG and curve fitting, Filtering notes the presence/absence of the artefact removal.

ANOVA Results	Area	Method	Filtering	Method*Filtering
Closed Eyes Relax	Occipital	0.112	0.084	0.830
	Parietal	0.025 *	0.989	0.831
	Frontal	0.057	0.061	0.831
Open Eyes Relax	Occipital	0.933	0.109	0.377
	Parietal	0.332	0.297	0.139
	Frontal	0.382	0.540	0.268
Closed Eyes Move 1	Occipital	0.104	0.049 *	0.090
	Parietal	0.104	0.525	0.301
	Frontal	0.146	0.588	0.320
Closed Eyes Move 2	Occipital	0.313	0.882	0.853
	Parietal	0.047 *	0.397	0.252
	Frontal	0.059	0.133	0.077
Closed Eyes Imaginary 1	Occipital	0.026 *	0.050 *	0.707
	Parietal	0.078	0.587	0.491
	Frontal	0.012 *	0.088	0.940
Closed Eyes Imaginary 2	Occipital	0.634	0.002 *	0.580
	Parietal	0.919	0.260	0.222
	Frontal	0.024 *	0.253	0.818
Open Eyes Move 1	Occipital	-	-	-
	Parietal	-	-	-
	Frontal	-	-	-
Open Eyes Move 2	Occipital	-	-	-
	Parietal	-	-	-
	Frontal	-	-	-
Open Eyes Imaginary 1	Occipital	0.191	0.924	0.070
	Parietal	0.013 *	0.372	0.580
	Frontal	0.821	0.168	0.070
Open Eyes Imaginary 2	Occipital	0.189	0.236	0.423
	Parietal	0.002 *	0.956	0.728
	Frontal	0.235	0.162	0.891

**Table 4:** The significantly different cases from Table 3. Most differences arose from the used peak determination method, but there are examples of the filtering-out of artefacts causing differences.

**Significant**		**Cl. Eyes Relax**		**Cl. Eyes Move**		**Cl. Eyes Imag.**		**Cl. Eyes Imag.**	
		**Parietal**		**Parietal**		**Occipital**		**Frontal**	
		*Mean*	*SD*	*Mean*	*SD*	*Mean*	*SD*	*Mean*	*SD*
**Method**	*Curve Fitting*	9.950	0.250	100.003	0.357	100.094	0.324	90.670	0.267
	*CoG*	90.780	0.222	90.904	0.359	90.985	0.305	90.473	0.247
		**Cl0. Eyes Imag0.**		**O0. Eyes Imag0.**		**O0. Eyes Imag0.**			
		**Frontal**		**Parietal**		**Parietal**			
		*Mean*	*SD*	*Mean*	*SD*	*Mean*	*SD*		
**Method**	*Curve Fitting*	90.716	0.328	100.325	0.328	100.457	0.385		
	*CoG*	90.505	0.269	90.972	0.325	100.210	0.402		
		**Cl0. Eyes Move**		**Cl0. Eyes Imag0.**		**Cl0. Eyes Imag0.**			
		**Occipital**		**Occipital**		**Occipital**			
		*Mean*	*SD*	*Mean*	*SD*	*Mean*	*SD*		
**Filtering**	*Unfiltered*	100.448	0.405	100.015	0.317	90.948	0.244		
	*Filtered*	100.307	0.413	100.064	0.311	100.042	0.246		

**Table 5:** Manual inspection of peaks from the BCI recordings (phase three of the experiment), when the stimuli were not flickering. Values in parenthesis mark cases when both the automatic and manual inspections found peaks, however, the peaks were more than 0.5 Hz apart, so they were considered wrong. The not parenthesized numbers in the same cells include these cases.

Manual Inspection		Manual Peaks			
		Unfiltered		Filtered	
		Peak Found	No Peak	Peak Found	No Peak
**Automatic Peaks**	**Peak Found**	594	86 (16)	781	43 (11)
	**No Peak**	243	939	182	856

**Table 6:** Manual inspection of peaks from the BCI recordings (phase three of the experiment), when the stimuli were flickering. Values in parenthesis mark cases when both the automatic and manual inspections found peaks, however, the peaks were more than 0.5 Hz apart, so they were considered wrong. The not parenthesized numbers in the same cells include these cases.

Manual Inspection		Manual Peaks			
		Unfiltered		Filtered	
		Peak Found	No Peak	Peak Found	No Peak
**Automatic Peaks**	**Peak Found**	893	183 (12)	1206	60 (8)
	**No Peak**	528	1882	600	1620

**Table 7:** Performance results of the online BCI tasks. The top row indicates the scenario.

	Relaxing			Move & No Filter			Move & Filter		
	Acc (%)	ITR (bpm)	Time (s)	Acc (%)	ITR (bpm)	Time (s)	Acc (%)	ITR (bpm)	Time (s)
**S1**	92.3	17.2	67.3	100.0	19.2	75.0	92.3	11.0	105.0
**S2**	100.0	24.5	73.5	100.0	19.6	91.8	94.1	15.8	102.0
**S3**	100.0	22.9	78.5	-	-	-	89.5	7.9	194.0
**S4**	100.0	24.0	120.0	100.0	23.2	124.0	93.3	16.3	170.0
**S5**	95.0	11.6	169.0	87.5	7.0	258.0	95.5	8.8	249.0
**S6**	100.0	23.8	90.8	-	-	-	-	-	-
**S7**	100.0	24.6	73.3	94.1	9.9	163.0	90.5	6.4	275.0
**S8**	100.0	13.2	136.0	94.7	15.0	123.0	93.8	12.5	120.0
**S9**	100.0	24.5	103.0	100.0	23.8	106.0	100.0	24.0	105.0
**S10**	94.1	17.5	92.5	100.0	22.4	80.3	100.0	21.9	82.3
**S11**	100.0	18.0	102.0	94.0	11.0	142.0	86.0	7.0	202.0
**S12**	94.1	16.2	99.8	100.0	13.8	130.0	80.0	5.4	267.0
**S13**	100.0	24.2	74.3	100.0	22.8	78.8	100.0	22.6	79.8
**S14**	100.0	23.8	75.5	94.1	12.4	130.0	94.1	14.8	109.0
**S15**	100.0	24.2	74.3	100.0	20.3	88.5	100.0	17.3	104.0
**Mean**	**98.37**	**20.68**	**95.32**	**97.26**	**16.96**	**122.34**	**93.51**	**13.69**	**154.58**
**SD**	**20.68**	**4.57**	**28.22**	**4.00**	**5.71**	**49.00**	**5.81**	**6.27**	**70.40**
